# Artificial Intelligence Increases the Agreement among Physicians Classifying Focal Skeleton/Bone Marrow Uptake in Hodgkin’s Lymphoma Patients Staged with [^18^F]FDG PET/CT—a Retrospective Study

**DOI:** 10.1007/s13139-022-00765-3

**Published:** 2022-08-19

**Authors:** May Sadik, Jesús López-Urdaneta, Johannes Ulén, Olof Enqvist, Per-Ola Andersson, Rajender Kumar, Elin Trägårdh

**Affiliations:** 1 Department of Molecular and Clinical Medicine, Clinical Physiology, Sahlgrenska University Hospital, Sahlgrenska Academy at the University of Gothenburg, Gothenburg, Sweden; 2 Eigenvision AB, Malmö, Sweden; 3 Department of Electrical Engineering, Chalmers University of Technology, Gothenburg, Sweden; 4 Department of Haematology, Södra Älvsborg Hospital, Borås, Sweden; 5 Institute of Medicine, Sahlgrenska Academy, Gothenburg University, Gothenburg, Sweden; 6 Department of Nuclear Medicine, Post Graduate Institute of Medical Education and Research, Chandigarh, India; 7 Clinical Physiology and Nuclear Medicine, Lund University and Skåne University Hospital, Malmö, Sweden

**Keywords:** Artificial intelligence, Hodgkin disease, Bone marrow, Observer variation, Fluorodeoxyglucose F18

## Abstract

**Purpose:**

Classification of focal skeleton/bone marrow uptake (BMU) can be challenging. The aim is to investigate whether an artificial intelligence–based method (AI), which highlights suspicious focal BMU, increases interobserver agreement among a group of physicians from different hospitals classifying Hodgkin’s lymphoma (HL) patients staged with [^18^F]FDG PET/CT.

**Methods:**

Forty-eight patients staged with [^18^F]FDG PET/CT at Sahlgenska University Hospital between 2017 and 2018 were reviewed twice, 6 months apart, regarding focal BMU. During the second time review, the 10 physicians also had access to AI-based advice regarding focal BMU.

**Results:**

Each physician’s classifications were pairwise compared with the classifications made by all the other physicians, resulting in 45 unique pairs of comparisons both without and with AI advice. The agreement between the physicians increased significantly when AI advice was available, which was measured as an increase in mean Kappa values from 0.51 (range 0.25–0.80) without AI advice to 0.61 (range 0.19–0.94) with AI advice (*p* = 0.005). The majority of the physicians agreed with the AI-based method in 40 (83%) of the 48 cases.

**Conclusion:**

An AI-based method significantly increases interobserver agreement among physicians working at different hospitals by highlighting suspicious focal BMU in HL patients staged with [^18^F]FDG PET/CT.

## Introduction

Skeleton/bone marrow involvement in newly diagnosed Hodgkin’s lymphoma (HL) patients is an important predictor of adverse outcome [1]. However, classifications of focal skeleton/bone marrow uptake (BMU) in [^18^F]FDG PET/CT images can be challenging. We previously performed a study involving 10 nuclear medicine physicians with various levels of experience in [^18^F]FDG PET/CT working at different hospitals. They were asked to interpret 48 consecutive, untreated HL patients staged with [^18^F]FDG PET/CT regarding focal and diffuse BMU. The results showed moderate agreement with mean Kappa values of 0.51 (range 0.25–0.80) and 0.41 (range 0.03–0.68), respectively [2]. In worst-case scenario, one physician could classify a patient as not having focal BMU, while the other classified the same patient as having focal uptake. With the intention of highlighting suspicious focal uptake in skeleton/bone marrow and reducing the risk of it being overlooked, we developed an AI-based method flagging suspicious uptake with red color in the [^18^F]FDG PET/CT images and automatically calculates a standardized uptake value (SUV) index for diffuse BMU (spine bone marrow SUV_median_/liver SUV_median_) [2].

Our aim was therefore to investigate whether the AI-based method, by highlighting suspicious focal uptake and presenting a quantitative value of diffuse BMU, increases interobserver agreement for both focal and diffuse BMU among a group of physicians from different hospitals classifying HL patients staged with [^18^F]FDG PET/CT.

## Methods

### Patients

The same patient material as a recent study was used [2]. All 49 patients who had undergone staging by [^18^F]FDG PET/CT between 2017 and 2018 at Sahlgrenska University Hospital, with biopsy-proven HL, were retrospectively included. The patients were newly diagnosed and untreated. One patient was excluded due a to falsely reported injection time. The final group consisted of 48 patients with a median age of 35 years (range 7–75) and 46% of the patients were female.

### Image Acquisitions

As in our recent study [2], PET/CT data were obtained using an integrated PET/CT system (Siemens Biograph 64 Truepoint). The adult patients were injected with 4 MBq/kg [^18^F]FDG (maximum 400 MBq) and fasted for at least 6 h prior to injection of FDG. The injected amount of radioactivity for children was according to the EANM Dosage Card (Version 5.7.2016). The standard accumulation time was 60 min. Images were acquired with 3 min per bed position from the base of the skull to the mid-thigh. PET images were reconstructed with a slice thickness of 5 mm and slice spacing of 3 mm with an iterative OSEM 3D algorithm (4 iterations and 8 subsets) and a matrix size of 168 × 168. CT-based attenuation and scatter corrections were applied. A low-dose CT scan (64-slice helical, 120 kV, 30 mAs, 512 × 512 matrix) was obtained covering the same part of the patient as the PET scan. The CT was reconstructed using a filtered back projection algorithm with a slice thickness and spacing matching those of the PET scan [2, 3].

### Image Interpretations

**Fig. 1:**
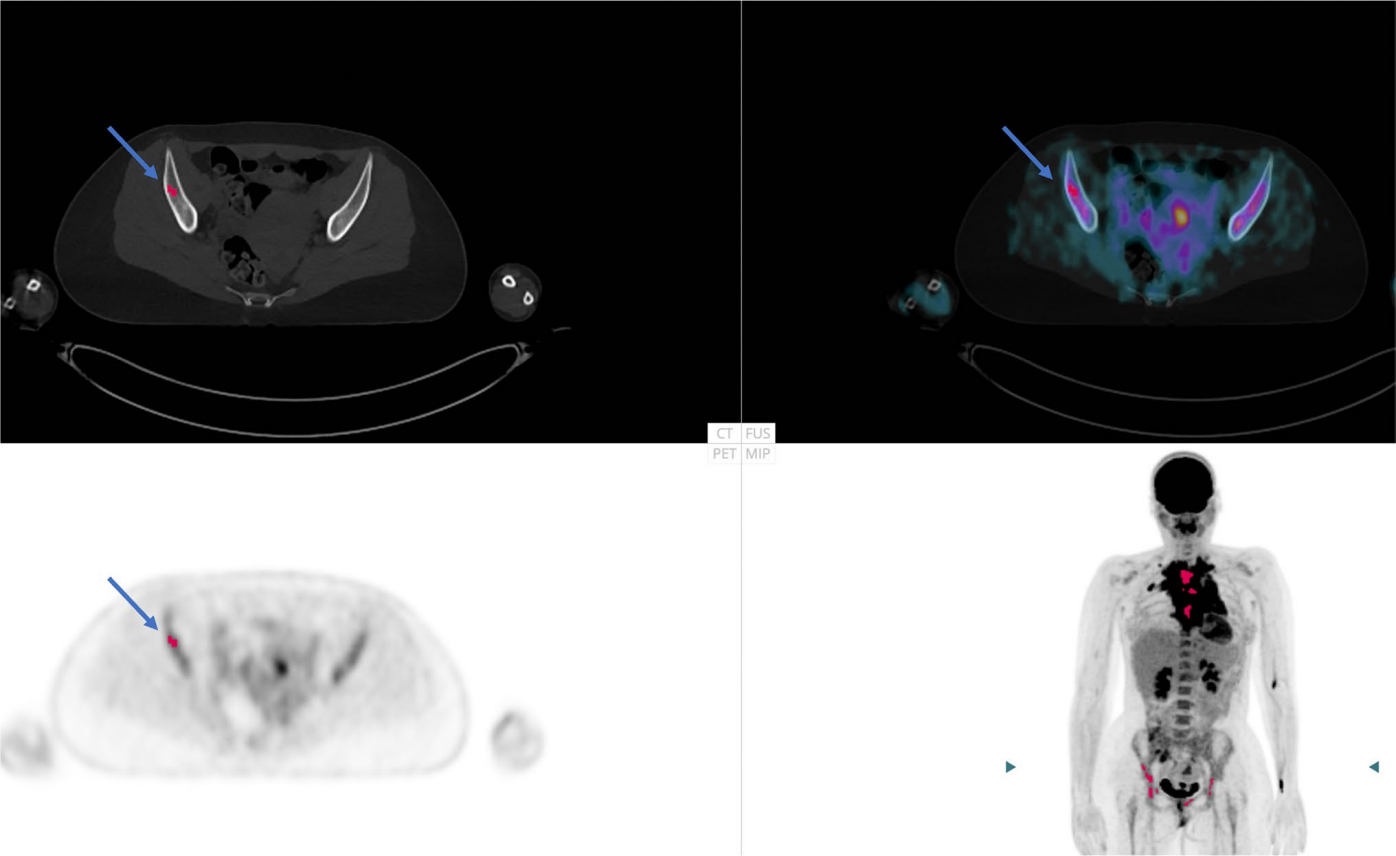
Example of a patient with focal skeletal/bone marrow uptake (highlighted with a red dot by artificial intelligence (AI), blue arrow) and increased diffuse bone marrow uptake (SUV_index_ 1.4). In this patient, 7 of the 10 physicians agreed with AI regarding focal bone marrow involvement

Ten nuclear medicine physicians with 2–12 years of experience in PET/CT working in three different hospitals (two in Sweden (Malmö/Lund and Gothenburg) and one in India (Chandigarh)) interpreted the images without AI advice 6 months before the present study [2]. The same physicians were asked to participate again, this time considering the computer advice, i.e., the AI-based method highlighted suspicious focal uptake in red and presented a median SUV_index_ (SUV_median_ spine bone marrow/SUV_median_ liver) (Fig. 1). Briefly, the SUV_index_ was calculated using the whole spine segmentation performed by a convolutional neural network (CNN) [4] (excluding 5 mm from the edges) [2] and the CNN-based whole liver segmentation (excluding 2 cm from the edges) [3]. A SUV_index_ value of > 1.0 indicates high BMU. The physicians separately classified the 48 [^18^F]FDG PET/CT images regarding diffuse and focal uptake in skeletal/bone marrow in the following four categories [1].

- Low diffuse bone marrow uptake and no focal lesion(s)
- Low diffuse bone marrow uptake and focal lesion(s)
- High diffuse bone marrow uptake and no focal lesion(s)
- High diffuse bone marrow uptake and focal lesion(s)

The red coloring of the AI focal uptake could easily be turned off/on by the observers. The cases were presented in different computer-generated randomized order for each physician. Information regarding sex and age was presented, and that the investigations involved untreated staging HL patients. The physicians were instructed to classify the cases as they normally do in the clinical setting. The review process was performed using RECOMIA software (recomia.org), and every case was presented with CT images, PET images, fused PET/CT images, and MIP images. The interpreter was also able to shift between sagittal, coronal, and transverse planes. The PET images could be displayed in different colors with the images scaled to an upper SUV threshold of 5, and the latter could also be changed. The CT images could be shifted to the skeleton window.

### Artificial Intelligence - Architecture

Detailed information regarding the development of the AI-based method, i.e., segmentation of the skeleton and liver and classifications of focal and diffuse uptake in the skeleton/bone marrow, can be found in [2–5]. Briefly, CNN was used to find the skeletal and liver anatomy, excluding 3–7 mm from the bone edges and 2 cm from the liver edges. Thereafter, mathematical rules were applied to identify focal and diffuse uptake in bones [2–5].

The study was approved by the Ethics Committee at Gothenburg University, and the need for written informed consent was waived (#2019-01274). We certify that the study was performed in accordance with the ethical standards laid down in the 1964 Declaration of Helsinki and its later amendments.

### Statistical Analyses

The Kappa coefficient (*K*) was used in the interobserver comparisons between physicians for the classifications of both focal skeletal/bone marrow uptake and diffuse BMU. Kappa takes into account chance agreement, and some suggested interpretation guidelines are as follows: values < 0 indicate no agreement; values between 0 and 0.20 indicate slight agreement; values between 0.21 and 0.40 indicate fair agreement; values between 0.41 and 0.60 indicate moderate agreement; values between 0.61 and 0.80 indicate substantial agreement; and values between 0.81 and 1 indicate almost perfect agreement [6]. Wilcoxon Signed-Rank with a significance level of 0.05, two-tailed, was used to test the difference in agreement between the classifications without and with the advice of the AI-based method.

## Results

### Focal Uptake

#### Physician vs Physician Without/with AI Advice

Kappa values were calculated to compare two physicians’ classifications of all the patients. Each physician could be compared to the other nine physicians, resulting in nine Kappa values. All together, 45 unique pairs of physicians with corresponding Kappa values were calculated (10 physicians × 9 comparisons = 90; comparing physicians A to physician B and physician B to physician A gives the same Kappa values; hence, only 45 comparisons are unique). The procedure was applied both to the first session without AI and to the second session with the AI advice.

**Fig. 2:**
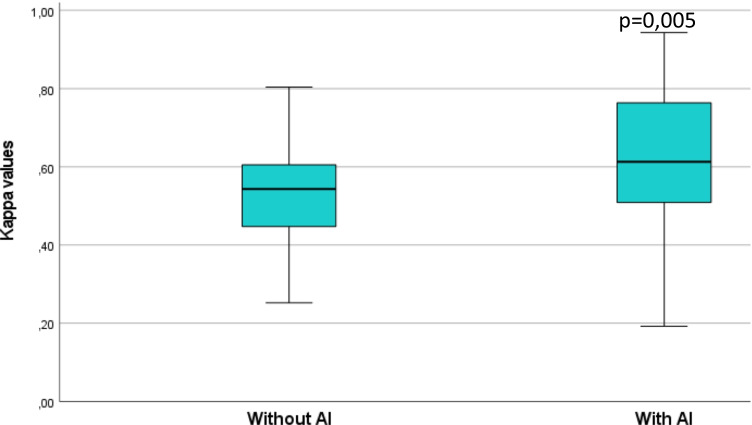
Box-plot showing Kappa values without/with artificial intelligence (AI) for the paired readers regarding the interpretations of focal uptake

The agreement between the physicians increased significantly when AI advice was available, measured as an increase in mean Kappa values from 0.51 (range 0.25–0.80) without AI advice to 0.61 (range 0.19–0.94) with AI advice (*p* = 0.005) (Fig. 2).

#### AI-Assisted Evaluation

Fourteen of the 48 cases were classified as having focal skeleton/bone marrow uptake by the AI-based method, while the majority of physicians (> 5 physicians of the totally 10) classified 7/48 as positive without AI advice, indicating that the AI-based method was adjusted towards high sensitivity to warn for suspicious uptake [2]. The high positive rate of the AI-based method did not influence the physicians to classify more cases as positive (92/480 positive classifications without the AI advice (10 physicians interpreting 48 cases = 480 classifications) versus 96/480 with the AI advice). All 7 cases classified as positive by the majority of physicians were identified as positive by the AI-based method.

In total, the majority of the physicians agreed with the AI-based method in 40 (83%) of the 48 cases. An example is shown in Fig. 1.

### Diffuse Bone Marrow Uptake

#### Physician vs Physician Without/with AI Advice

The agreement between the physicians regarding diffuse BMU did not change significantly when AI SUV_index_ was made available. The mean Kappa values of the pairwise comparisons between the physicians were 0.41 (range 0.03–0.68) without AI SUV_index_ and 0.44 (range 0.11–0.91) with AI SUV_index_ (*p* = 0.79).

#### AI-Assisted Evaluation

**Fig. 3:**
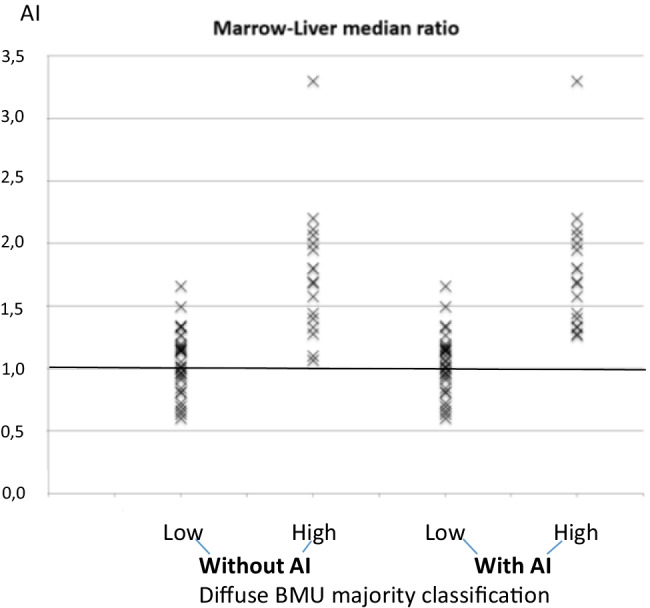
SUV index: (spine marrow uptake/liver) as calculated by the artificial intelligence–based method (AI) compared with the physicians’ majority classifications of diffuse bone marrow uptake (BMU)

The automatic AI calculations of SUV_index_ indicated that 32 of the totally 48 cases had an index > 1.0 (high diffuse BMU), while the majority of the physicians (> 5 physicians of the totally 10) classified 17 patients as having high diffuse BMU with AI advice. Figure 3 shows the physicians’ majority classifications of diffuse BMU without and with AI advice.

In 9 (28%) of the 32 cases, most physicians (> 5 physicians of the totally 10) classified them as having low diffuse BMU, despite that the automated AI advice suggested high diffuse BMU (i.e., an SUV_index_ ranging between 1.1 and 1.3). Quality control was performed by manual region of interest index calculations (L3/L4 vertebrae SUV_median_/liver SUV_median_) showing an SUV_index_ ranging between 1.1 and 1.4, i.e., diffuse BMU slightly above the index of 1.0 [2]. The regions L3/L4 and the upper right part of the liver were chosen regarding to the recommendations by [1].

## Discussion

Our results showed that it is possible to increase agreement between physicians with various levels of experience working at different hospitals by using an AI-based method that highlights suspicious focal skeleton/bone marrow uptake in HL patients staged with [^18^F]FDG PET/CT. The physicians’ agreement regarding focal uptake changed significantly (*p* = 0.005) from being moderate (*K* = 0.51) without using the AI-based method to substantial (*K* = 0.61) using the software in the classification of the same 48 cases, interpreted twice with 6 months apart (Fig. 2). Machine-based detection is being developed in a variety of medical fields for the purpose of highlighting suspicious pathological findings, reducing the amount of time spent for segmentation of for example total lesion volume and for increasing agreement among colleagues [7–11]. To our knowledge, no AI-based method has yet been presented for the detection of focal skeleton/bone marrow uptake in HL patients, thus making it difficult to directly compare with other studies.

We also showed that this AI-based method objectively provides information regarding high versus low BMU by calculating the SUV_median_ in the spine marrow and the liver [2]. However, no significant change in agreement was observed between the physicians in the classifications of diffuse BMU. The mean Kappa coefficient remained moderate both without (0.41) and with (0.44) the advice of the AI-based method.

The reason we chose the median SUV in the SUV_index_ calculations is for the purpose to exclude extreme values, for example, in patients having focal uptake in bone marrow and/or the liver. Quality control was also performed (by manually drawing region of interest in L3/L4 and the upper right part of the liver) and we compared the findings with the automatic AI SUV_index_ calculations (including the whole spine and the liver). The results for the two approaches were comparable [2].

A favorable method to present the correctness of the classifications is to obtain a gold standard for each focal and diffuse skeleton/bone marrow uptake in the PET/CT images and present the accuracy, for example, by calculating the sensitivity and specificity. However, bone marrow and skeleton biopsies in each suspicious uptake and in each patient are often difficult to obtain and the second-best approach may be to compare the physicians’ classifications with each other, thereby reflecting their precision. We invited 10 physicians with various levels of experience (2–12 years) in interpreting PET/CT images working in three different hospitals and compared their interpretations pairwise with each other. It is already known that experienced physicians working at the same institution, having continuous discussions, tend to agree more in the interpretations [12, 13]. However, every day clinical work differs from this perfect setting. It is not uncommon that less experienced physicians interpret FDG-PET/CT images that is why we feel that our results are generalizable. We present here a complementary approach to lower the variability in the interpretations.

We also welcome future collaboration with other research groups to further test the AI-based method both for focal and diffuse BMU by including new HL patients staged with PET/CT, investigated with other cameras and in other hospitals.

**Fig. 4:**
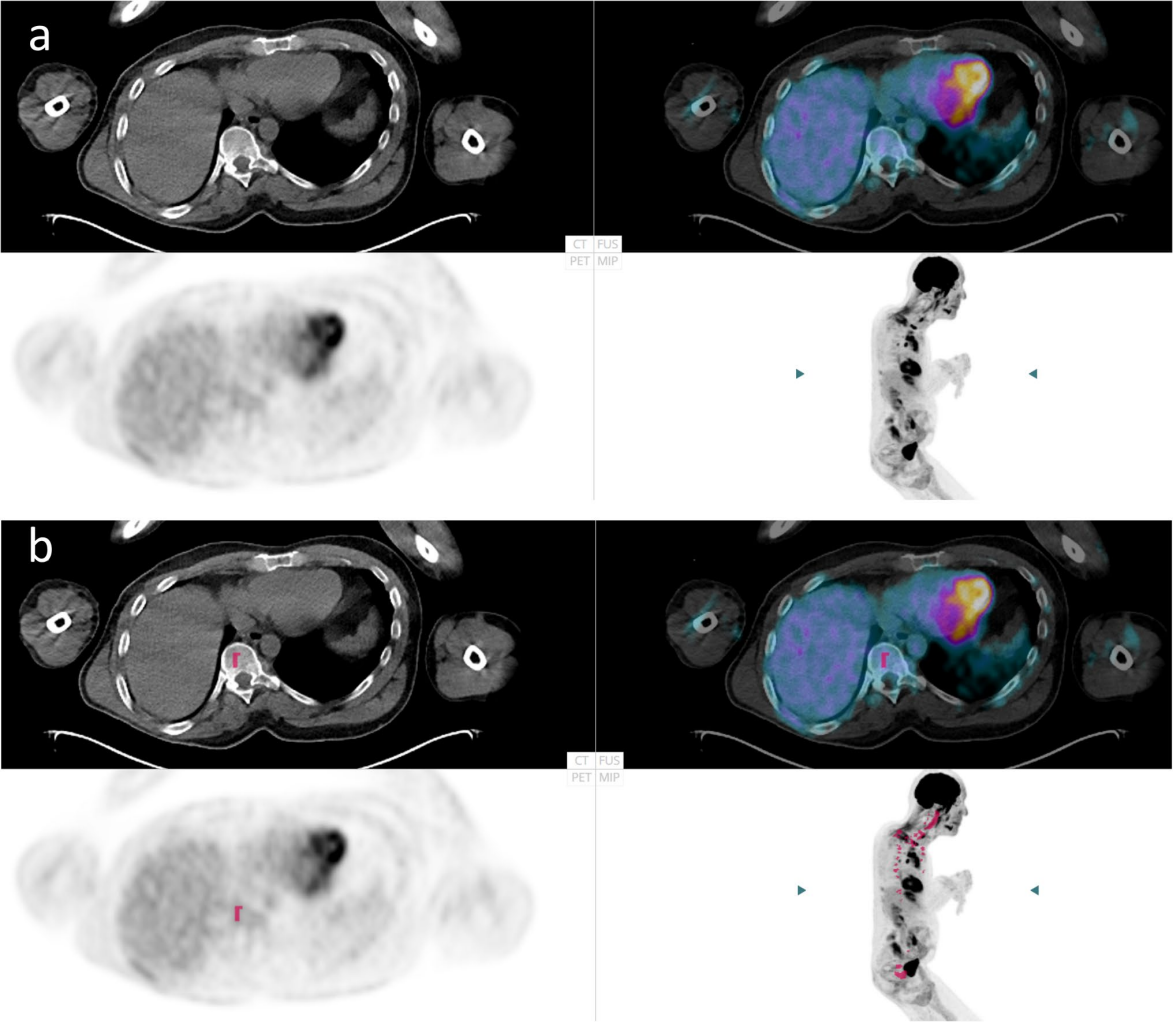
The same patient examination is shown in **a** (without) and **b** (with artificial intelligence (AI). This is an example of a false positive classification by AI. Nine of the 10 physicians classified this case as not having focal skeletal involvement both without and with AI

Pedersen et al. emphasized the importance of finding focal bone lesions (uni- or multifocal) because both the progression-free survival rate and overall survival significantly indicate poorer prognosis compared with the nonbone lesion group [1]. The idea of AI is to focus the physician’s attention on suspicious uptake. We have developed the software to present a higher positive rate. Our results showed that the AI-based method did not influence the physicians to classify more cases as positive (Fig. 4) but rather that it improved their agreement (Fig. 2). Interpretations of medical images from the same patient should ideally be the same regardless of physician experience or hospital setting. All seven cases classified as positive by the majority of physicians (> 5 physicians of the totally 10) were identified as positive by the AI-based method. However, the final decision should always be made by a human observer, and computer software should never be held responsible.

The strength of human observers is their ability to recognize rare findings, while the advantage of computer analysis is rapid mathematical calculations, such as comparing spine bone marrow with the liver, the SUV_index_. The pair of physicians with the lowest agreement in the classifications of diffuse BMU had a Kappa value of 0.11, indicating limited agreement. Thus, former authors’ findings [1] are in concordance with ours that the most common cause of disagreement between the physicians and AI was due to the underestimation of the diffuse BMU (Fig. 3). Indeed, it seems to be rather difficult to classify high BMU when the SUV_index_ is slightly above 1.0 that is why an objective presentation could be more preferable. In various lymphomas, total metabolic tumor volume (MTV) and total lesion glycolysis (TLG) have emerged as promising biomarkers of outcome [14]. Diffuse increased uptake in the spleen is one of four parameters proposed to be included in the quantification of MTV and TLG [14], if the spleen uptake is greater than the liver and in the absence of reactive changes in bone marrow (i.e., SUV_index_ equal to or < 1.0). The presentation of an automatic AI-SUV_index_ could facilitate the decision whether or not diffuse spleen uptake should be included in MTV and TLG. Therefore, we calculate SUV_index_ both for bone marrow/liver and in the future, also SUV_index_ for the spleen/liver.

## Conclusions

An AI-based method significantly increases interobserver agreement among physicians working at different hospitals by highlighting suspicious focal skeleton/bone marrow uptake in HL patients staged with [^18^F]FDG PET/CT. No significant impact on agreement was found among physicians in the classification of diffuse BMU.

## Data Availability

The datasets generated and/or analyzed during the current study are not publicly available due to ethical considerations.
